# A novel HIV-1-encoded microRNA enhances its viral replication by targeting the TATA box region

**DOI:** 10.1186/1742-4690-11-23

**Published:** 2014-03-12

**Authors:** Yijun Zhang, Miaomiao Fan, Guannan Geng, Bingfeng Liu, Zhuoqiong Huang, Haihua Luo, Jie Zhou, Xuemin Guo, Weiping Cai, Hui Zhang

**Affiliations:** 1 Institute of Human Virology, Zhongshan School of Medicine, Sun Yat-sen University, Guangzhou, Guangdong 510080, China; 2 Key Laboratory of Tropical Disease Control of Ministry of Education, Zhongshan School of Medicine, Sun Yat-sen University, Guangzhou, Guangdong 510080, China; 3 Department of Infectious Diseases, Guangzhou Eighth People’s Hospital, Guangzhou Medical University, Guangzhou, Guangdong 510080, China

**Keywords:** HIV-1 viruses, Viral miRNA, TATA box, Transcription activation, Viral replication, Latency

## Abstract

**Background:**

A lot of microRNAs (miRNAs) derived from viral genomes have been identified. Many of them play various important roles in virus replication and virus-host interaction. Cellular miRNAs have been shown to participate in the regulation of HIV-1 viral replication, while the role of viral-encoded miRNAs in this process is largely unknown.

**Results:**

In this report, through a strategy combining computational prediction and deep sequencing, we identified a novel HIV-1-encoded miRNA, miR-H3. MiR-H3 locates in the mRNA region encoding the active center of reverse transcriptase (RT) and exhibits high sequence conservation among different subtypes of HIV-1 viruses. Overexpression of miR-H3 increases viral production and the mutations in miR-H3 sequence significantly impair the viral replication of wildtype HIV-1 viruses, suggesting that it is a replication-enhancing miRNA. MiR-H3 upregulates HIV-1 RNA transcription and protein expression. A serial deletion assay suggests that miR-H3 targets HIV-1 5′ LTR and upregulates the promoter activity. It interacts with the TATA box in HIV-1 5′ LTR and sequence-specifically activates the viral transcription. In addition, chemically-synthesized small RNAs targeting HIV-1 TATA box activate HIV-1 production from resting CD4^+^ T cells isolated from HIV-1-infected patients on suppressive highly active antiretroviral therapy (HAART).

**Conclusions:**

We have identified a novel HIV-1-encoded miRNA which specifically enhances viral production and provide a specific method to activate HIV-1 latency.

## Background

MiRNA represents a class of small RNA ranged from 21-24 nts, which plays important regulatory roles in animal, plant, and fungi [1,2]. Virus-encoded miRNAs were initially identified from Epstein-Barr viruses [3]. Since then, increasing virus-encoded miRNAs have been identified [4,5]. Most of these miRNAs were reported in DNA viruses such as Herpes and Polyoma viruses, but rarely in RNA viruses [5]. Because of the rapid development of deep sequencing technology which is much more sensitive and quantitative than the conventional cDNA clone sequencing method, more RNA virus-derived miRNAs have been discovered especially from HIV-1, WNV and BLV [6-9].

Most miRNAs repress gene expression through targeting the 3′ UTR of mRNA in cytoplasmic RISC for translation repression or mRNA degradation [1,10-12]. It has been revealed that 5′ UTR and exons could also be the targets of miRNAs for translation repression [13,14]. In addition, miRNAs could also enter the nucleus and modulate gene expression at transcriptional level [15-17]. These findings reveal multiple action modes are exploited by miRNAs for gene expression regulation.

MiRNAs play important roles in the interaction between parasites and their hosts. Cellular miRNAs could affect the viral replication, latency and mediate antiviral defense. For example, miR-122 that is enriched in the liver plays a key role in the accumulation of viral RNAs of hepatitis C viruses [18]. A cellular miRNA effectively restricts the accumulation of the retrovirus primate foamy virus type 1 (PFV-1) in human cells [19]. Our group reported that several miRNAs from resting human CD4^+^ T cells repress the translation of viral proteins and contribute to the latency of HIV-1 [20]. Conversely, viral miRNAs could facilitate viral infection through reducing the viral antigens or impairing the host antiviral immune response. For instances, SV40 miR-S1 down-modulates the production of the viral T antigen (TAg), an early protein which is not required during late infection [21]. An EBV-encoded miRNA miR-BART5 targets the proapoptotic factor PUMA to promote host cell survival [22]. Furthermore, an hCMV miRNA, miR-UL112-1, was reported to inhibit the expression of the stress-induced ligand MICB and enable hCMV to escape from the immune surveillance by NK cells [23].

It has been reported that human immunodeficiency virus type 1 (HIV-1) also encode several miRNAs and other small RNAs. Bennasser et al. first performed a computational prediction on HIV-1 encoded miRNAs and found five pre-miRNAs candidates [24]. Subsequently, several groups identified HIV-1 encoded miRNAs from the *nef* or the TAR element [25-28]. Through the new generation sequencing method, a number of HIV-1-encoded small RNAs were discovered, some of which exhibit the features of miRNA or small interfering RNA (siRNA) [7,29].

These HIV-1 derived small RNAs have been shown to modulate the cellular and/or viral gene expression. A *nef*-derived miRNA-miR-N367 could block HIV-1 Nef expression *in vitro*[28]. The expression of the TAR derived miRNA could protect the infected cells from apoptosis by down-regulating cellular genes involved in apoptosis [27,30]. Since the expression levels of small non-coding RNAs generated from RNA viruses are relatively low, their roles in viral replication remain largely elusive. Here, we initiated our project by searching for new HIV-1-derived miRNA(s) and have identified a surprising new function for a miRNA isolated from the reverse transcriptase sequence.

## Results

### Computational prediction of HIV-1-encoded miRNAs

**Figure 1:**
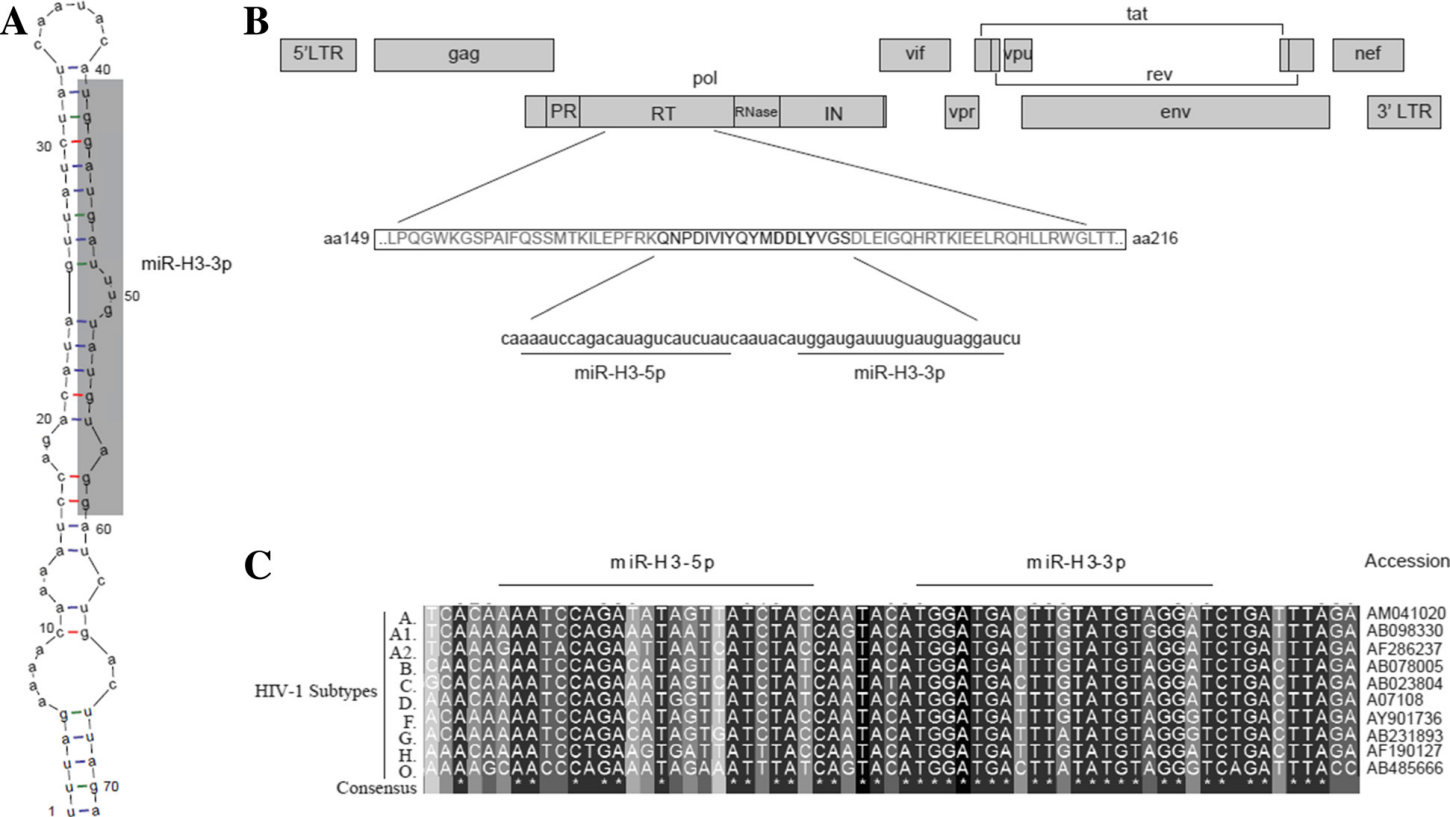
**Computational prediction of a novel HIV-1-encoded miRNA. (A)** The predicted secondary structure of miR-H3 precursor. **(B)** A schematic diagram of miR-H3 in the HIV-1 genome (start at 520 bp from the 5′ end of RT gene). **(C)** Conservation analysis of miR-H3 precursor among different HIV-1 subtypes. The accession number is listed beside each sequence.

By experimental methods, the architecture and secondary structure of the entire HIV-1 RNA genome have been clarified [31], which makes the prediction of HIV-1-encoded miRNA(s) more accurate. With the online software mireval (http://mimirna.centenary.org.au/mireval/), we predicted nine putative HIV-1-encoded miRNAs including the TAR region-derived miRNA which was reported previously (data not shown) [26]. However, in addition to TAR region-derived miRNA, only the precursor sequence of miR-H3 could form the classic stem-loop structure of miRNA according to the authentic secondary structure of HIV-1 RNA (Figure 1A and Additional file 1: Figure S1). The precursor sequence of miR-H3 is located at the coding sequence of the HIV-1 reverse transcriptase (RT), amino acids (a.a.) 174-191 (Figure 1B). The miR-H3-3p mature sequence covers the sequence of the RT catalytic domain (a.a. DDLY), which is highly conserved among different HIV-1 subtypes (Figure 1C).

### Experimental validation of miR-H3

**Figure 2:**
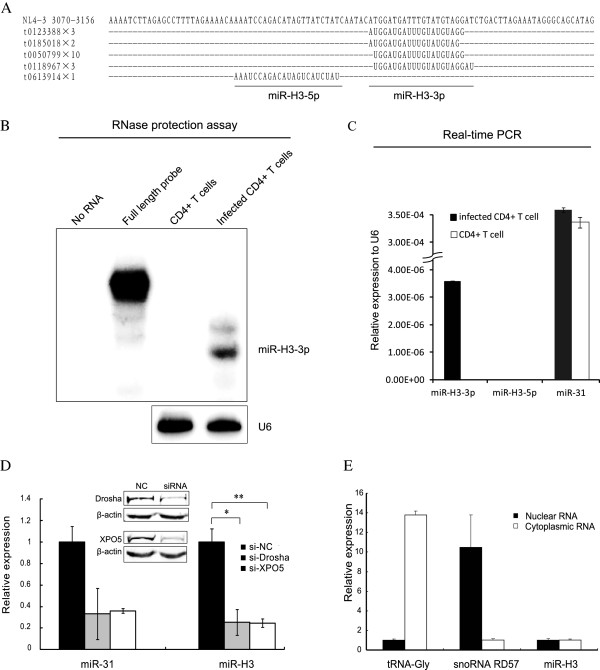
**Experimental validation of miR-H3. (A)** Mature miRNA sequences revealed by deep sequencing. Primary human CD4^+^ T-lymphocytes isolated from healthy donors were activated with anti-CD3(1 μg/ml) and anti-CD28(5 μg/ml) antibodies and infected with wildtype HIV-1_NL4-3_ viruses. The total RNAs from infected or uninfected CD4^+^ T-lymphocytes were extracted for deep sequencing on small RNA profiling. **(B)** RNase protection assay of small RNAs (<200 nt) isolated from primary CD4^+^ T-lymphocytes infected with wild type HIV-1_NL4-3_ viruses or uninfected CD4^+^ T-lymphocytes. The probe is corresponding to 34-71 nt of the miR-H3 precursor. U6 RNA was detected as the loading control for RPA. **(C)** Real-time qRT-PCR was conducted on total RNAs isolated from infected or uninfected activated CD4^+^ T-lymphocytes with primers for miR-H3-3p and miR-H3-5p respectively. MiR-31 was set as a positive control, while U6 RNA expression was set as the internal control and set as 1 unit. **(D)** Two key factors in miRNA processing, DROSHA and XPO5, were knocked-down with siRNAs, and the expression of miR-H3-3p from its precursor transfected into HEK293T was investigated by qRT-PCR as described above. **(E)** Subcellular localization of miR-H3. HEK293T cells were transfected with pCMV-ΔR8.2 vector. Fouty-eight hrs later, nuclear and cytoplasmic RNAs were isolated separately and the expression of tRNA-Gly, snoRNA-snoRD57 and miR-H3-3p were tested with real-time qRT-PCR. *P*-values were calculated using the two tailed unpaired Student’s t-test with equal variances, n = 3. *p < 0.05, **p < 0.01, ***p < 0.001.

To validate the expression of this miRNA, activated human primary CD4^+^ T-lymphocytes were infected with wildtype HIV-1_NL4-3_ viruses. The deep sequencing on small RNAs was conducted with total RNAs isolated from infected or uninfected CD4^+^ T-lymphocytes. Eighteen and one reads were found to correspond to 3p and 5p mature sequence of miR-H3 respectively (Figure 2A), which suggests miR-H3-3p (miR-H3 in the following text for convenience) is the guiding strand and miR-H3-5p is the passenger strand. TAR-derived miRNA has the similar reads in our deep sequencing data as the miR-H3-3p (data not illustrated). The expression levels of HIV-1-derived small RNAs are consistent with a recent report [7]. However, miR-H3-3p was not described in the previous studies, which may result from the different programs used for miRNA prediction or different sample preparing methods for deep sequencing [4,7,29,32]. To confirm the expression of miR-H3-3p, RNase protection assay (RPA), real-time qRT-PCR and primer extension assay were conducted. In CD4^+^ T-cells infected with wildtype HIV-1_NL4-3_, a small RNA was specifically detected by RPA with a ^32^P-labeled probe complementary to miR-H3-3p (Figure 2B). The lower band corresponded to the major fraction of the mature miR-H3-3p sequence of 19 nts, and the upper band possibly corresponded to the longer slicing products from the miR-H3 precursor, as suggested by the deep sequencing data. These results were also confirmed by real-time qRT-PCR assay on the total RNAs from HIV-1 virus-infected primary CD4^+^ T lymphocytes (Figure 2C) and primer extension assay (Additional file 1: Figure S2).

To further reveal whether the generation of miR-H3 is dependent on the miRNA processing pathway, the proteins required for miRNA precursor processing and transport such as Drosha and Exportin-5 were knocked down by siRNAs, and the expression of miR-H3-3p was significantly reduced (Figure 2D). The subcellular distribution analysis of miR-H3-3p suggested that it has equal amount in the nucleus and the cytoplasm, indicating a possible role it plays in the nucleus (Figure 2E).

### MiR-H3 enhances viral production and replication

**Figure 3:**
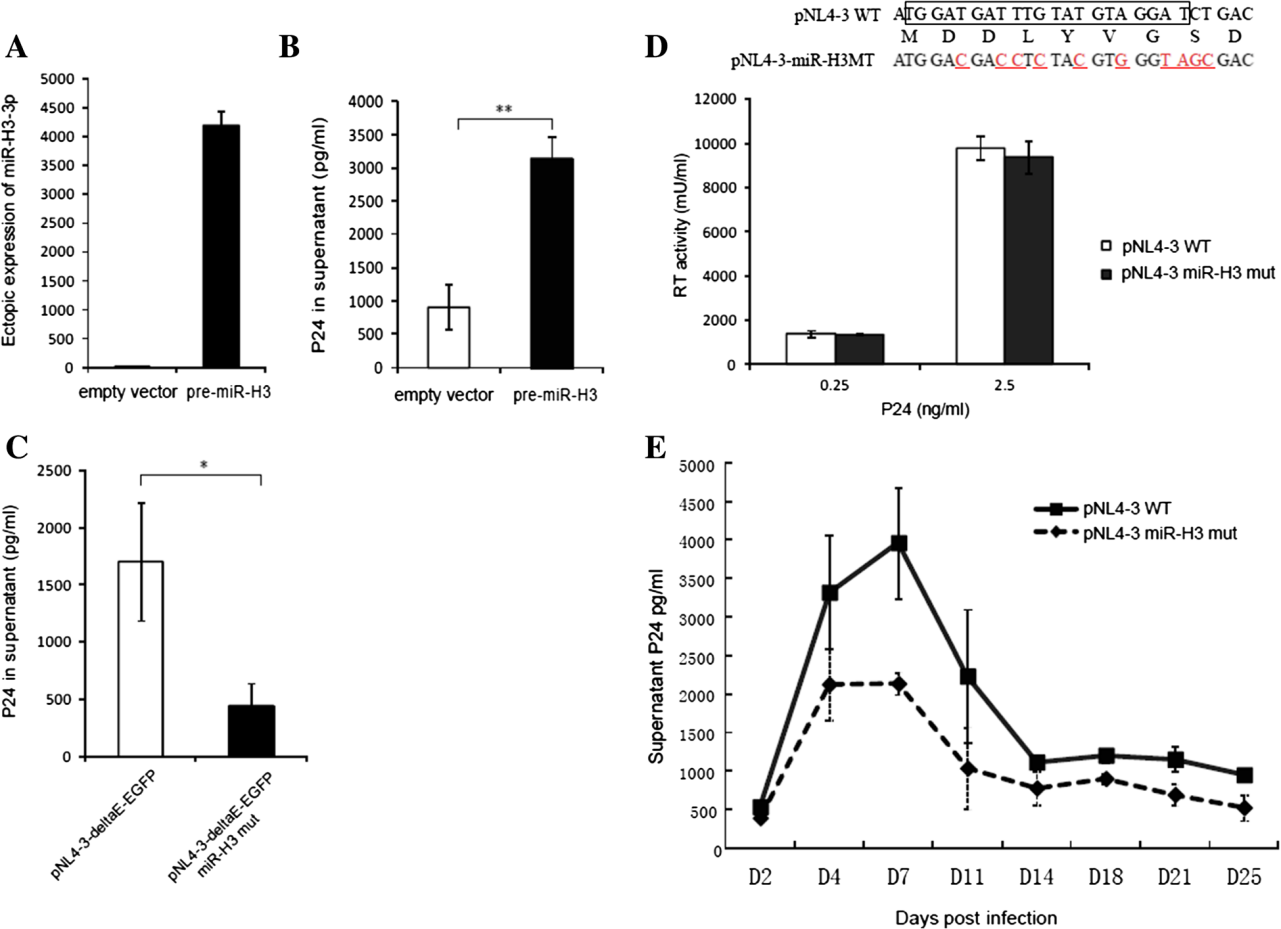
**Effect of miR-H3 on HIV-1 viral replication. (A)** Ectopic expression of miR-H3 by a construct containing its precursor. Mature miR-H3-3p sequence was tested with Real-time qPCR, an empty vector was transfected as control. **(B)** HEK293T cells were co-transfected with an Env-defective HIV-1 clone named pNL4-3-deltaE-EGFP and a miR-H3 precursor (pre-miR-H3) or the empty vector pEGFP-C1. The productions of P24 in the supernatant were determined at 48 hrs post-transfection. **(C)** HEK293T cells were transfected with pNL4-3-deltaE-EGFP or a miR-H3 mutant named pNL4-3-deltaE-EGFP miR-H3-mut. The productions of P24 in the supernatant were determined as described above. **(D)** Silent mutations were introduced to miR-H3 precursor (labeled in red with underline) to eliminate its processing without altering the amino acids of the HIV-1 RT gene (top). The reverse transcriptase activity of wildtype or miR-H3 deficient viruses was tested at different concentrations (bottom). **(E)** Equal amount (5 ng of P24 antigen) of wildtype and mutated viruses were used for the infection of 2x10^6^ activated primary CD4^+^ T-lymphocytes. The viral production in the supernatant was assayed by P24 ELISA at several time intervals. *P*-values were calculated using the two tailed unpaired Student’s t-test with equal variances, n = 3. *p < 0.05, **p < 0.01.

To investigate the possible effect of miR-H3 on viral replication, we generated a construct containing the precursor of miR-H3 which could express miR-H3 efficiently (Figure 3A). Mutations introduced to the precursor sequence significantly impaired the generation of mature miR-H3-3p (Additional file 1: Figure S3), suggesting the wildtype sequence is important for the proper processing of the precursor. The overexpression of miR-H3 substantially enhanced the virus production when the Env-defective HIV-1 clone, pNL4-3-deltaE-EGFP [33], was transfected into HEK293T cells (Figure 3B). This result was also confirmed with the overexpression of miR-H3 in a cell line named TZM-bl which contains an integrated HIV-1 promoter-driven luciferase gene [34] (Additional file 1: Figure S4). Alternatively, when the precursor sequence of miR-H3 was mutated to disturb its normal secondary structure without changing the corresponding amino acids in pNL4-3-deltaE-EGFP (Additional file 1: Figure S5A), the viral production was reduced (Figure 3C). To study the effect of miR-H3 on the replication of wildtype HIV-1 viruses, we introduced the similar silent mutations into the sequence of miR-H3-3p in the wildtype HIV-1_NL4-3_ to eliminate its precursor processing (Figure 3D top, Additional file 1: Figure S5B). The reverse transcriptase activity assay indicated that the mutations did not impair the activity of reverse transcriptase at different concentrations (Figure 3D bottom). The infectivity experiment of wildtype HIV-1 viruses suggested that the deficiency of miR-H3 substantially reduced the replication of HIV-1 in activated human CD4^+^ T-lymphocytes (Figure 3E).

### MiR-H3 increases viral RNA accumulation

**Figure 4:**
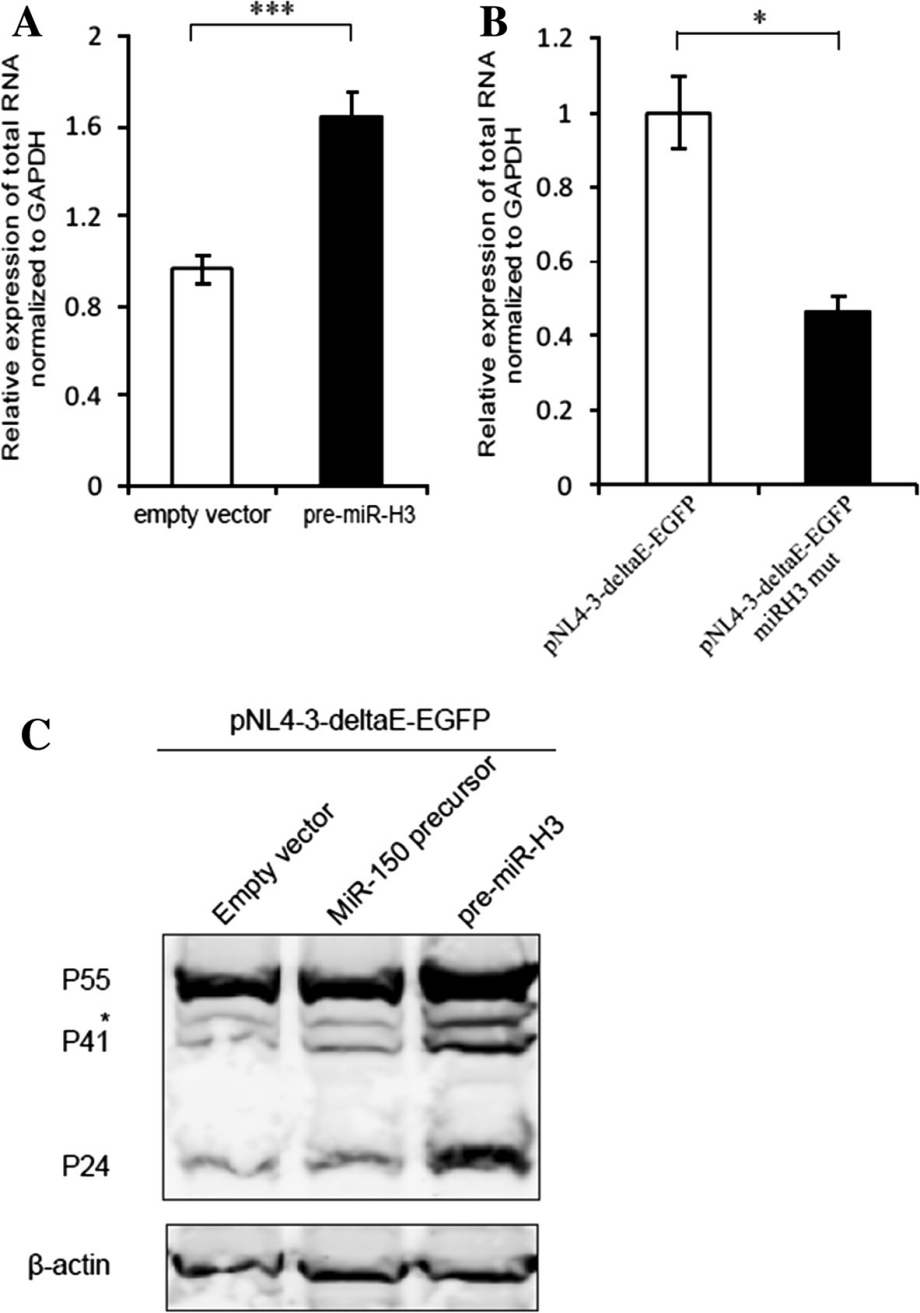
**MiR-H3 upregulates HIV-1 total RNA and protein level. (A)** Real-time qRT-PCR analysis of HIV-1 total transcripts from HEK293T cells transfected with pNL4-3-deltaE-EGFP and a construct harboring the miR-H3 precursor or the empty vector. **(B)** pNL4-3-deltaE-EGFP or a miR-H3-defective construct pNL4-3-deltaE-EGFP-miRH3-mut was transfected into HEK293T cells and total HIV-1 transcripts were assayed with real-time qRT-PCR at 48 hrs post-transfection. GAPDH was set as the internal control. **(C)** Western blot of HIV-1 structural protein P55, P41 and P24 in HEK293T cells transfected with pNL4-3-deltaE-EGFP and the empty vector, the construct containing miR-150 precursor or miR-H3 precursor. The β-actin was used as an internal control. *P*-values were calculated using the two tailed unpaired Student’s t-test with equal variances, n = 3. *p < 0.05, ***p < 0.001.

Since we observed miR-H3 could enhance virus production of the Env-defective HIV-1 clone pNL4-3-deltaE-EGFP, whose virus production begins with transcription, we firstly checked whether the transcription of HIV-1 total RNA was manipulated by miR-H3. Overexpression of miR-H3 by the precursor construct upregulated the expression of HIV-1 total RNAs transcribed from pNL4-3-deltaE-EGFP plasmid (Figure 4A). The HIV-1 total RNA is determined by the RNA transcripts containing the HIV-1 R and U5 sequence [35]. Alternatively, when the miR-H3-encoding sequence was mutated in the pNL4-3-deltaE-EGFP plasmid, there was a significative reduction of HIV-1 total RNAs transcribed from pNL4-3-deltaE-EGFP (Figure 4B). We next checked whether the HIV-1 protein production had been affected by miR-H3. Western blot was performed using an antibody against P24 protein which could also recognize its precursors P55 and P41 in the cytoplasm. The expression of Gag proteins including P55, P41 and P24 were substantially enhanced by miR-H3 (Figure 4C). These results suggested that miR-H3 could facilitate the accumulation of HIV-1 RNAs in the cells. This regulation could be achieved via two ways: one is by enhancing the transcriptional activity of the promoter, and another is by increasing the stability of the RNAs.

### MiR-H3 targets HIV-1 5′ LTR for promoter activation

**Figure 5:**
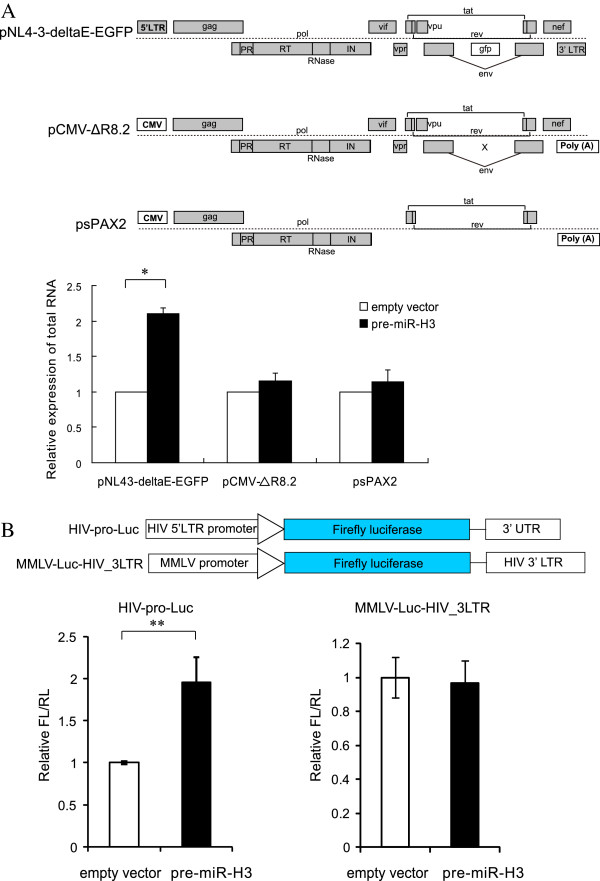
**MiR-H3 targets HIV-1 5′ LTR and upregulates HIV-1 promoter activity. (A)** Effects of miR-H3 overexpression on different HIV-1 derived lentiviral vectors. pNL4-3-deltaE-EGFP, pCMV-ΔR8.2 and pAX2 are all HIV-1 derived lentiviral vectors with major difference in their 5′ and 3′ ends: pNL4-3-deltaE-EGFP contains HIV-1 LTRs, the other two contain most viral protein genes which were driven by a CMV promoter. MiR-H3 precursor was co-transfected with these vectors separately, and then the HIV-1 total transcripts from these plasmids were determined by real-time qRT-PCR. **(B)** Dual-Luciferase assay was performed on the reporter construct with a HIV-1 5′-LTR functioning as the promoter or a HIV-1 3′-LTR sequence as the 3′-UTR region after co-transfection with the miR-H3 precursor or the empty vector. *P*-values were calculated using the two tailed unpaired Student’s t-test with equal variances, n = 3. *p < 0.05, **p < 0.01.

To identify the targeting site of miR-H3 on the HIV-1 genome, different generations of lentiviral vectors, which are derived from the HIV-1 genome with reduced viral genes and elements, were used to examine the possible direct target of miR-H3. The first is pNL43-deltaE-EGFP vector, which contains the entire HIV-1 genome except for the *env* gene which was impaired and replaced with a *gfp* gene. The second is pCMV-ΔR8.2 vector, which contains similar genes with pNL4-3-deltaE-EGFP but lacks of 5′ and 3′ LTR regions. The third is psPAX2 vector, which only contains *gag, pol, tat, rev* genes and *rre* motifs (Figure 5A top). When co-transfected these vectors with miR-H3 precursor or the empty vector, we found that miR-H3 could only enhance the RNA expression of pNL4-3-deltaE-EGFP, but not that of the other two vectors (Figure 5A bottom), suggesting its targeting site is located on 5′ or 3′ LTR region. To clarify which region is the target of miR-H3, the LTR regions were cloned into a luciferase reporter plasmid, pMIR- REPORT. The 5′ LTR sequence was inserted into the upstream of firefly luciferase gene to replace its CMV promoter, while the 3′ LTR sequence was inserted to the 3′ UTR region of the firefly luciferase gene with a MMLV (moloney murine leukemia virus) promoter whose activity is similar to that of HIV-1 5′LTR. Ectopic expression of miR-H3 substantially enhanced the luciferase activity of the construct containing HIV-1 5′ LTR as the promoter, but not that of the construct containing HIV-1 3′ LTR as the 3′-UTR (Figure 5B). These results implied that miR-H3 targets the 5′ LTR region of HIV-1 and most probably worked through enhancing the promoter transcriptional activity.

### MiR-H3 targets HIV-1 TATA box sequence-specifically

**Figure 6:**
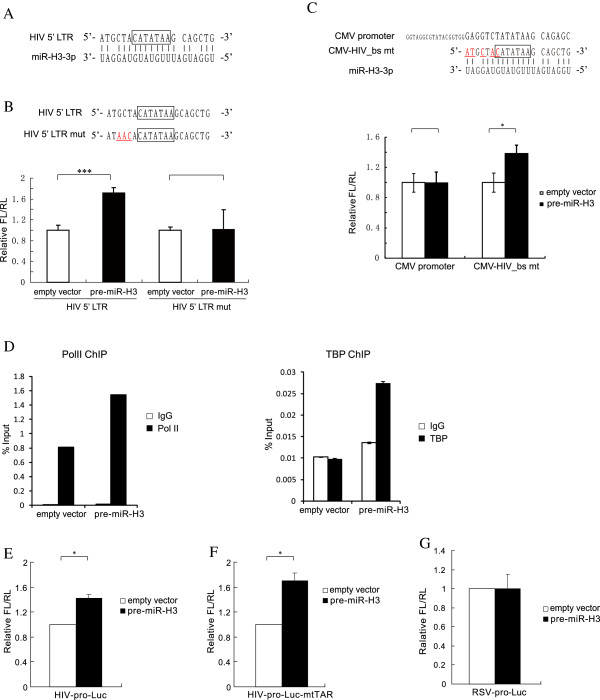
**MiR-H3 targets the TATA box in HIV-1 5′ LTR via sequence specific manner. (A)** Predicted binding site of miR-H3 in HIV-1 5′ LTR with the TATA box highlighted. The MFE of this interaction is -19.2 kcal/mol. **(B)** Mutations (labeled in red with underline) were introduced to the promoter, named HIV 5′ LTR mut. The effects of miR-H3 on promoter activity of wildtype or mutated promoter were determined with dual-luciferase assay. The TATA box motif was indicated with a box. **(C)** Top, the TATA box motif of CMV promoter was replaced by the binding site of miR-H3 on HIV-1 promoter (CMV-HIV_bs mt). The mutated nucleotides are indicated in red with underline. Bottom, the effects of miR-H3 on indicated promoter activities were determined with Dual-Luciferase assay as described above. The TATA box motif was indicated with a box. **(D)** ChIP assay of HEK239T cells co-transfected with pNL4-3-deltaE-EGFP and pre-miR-H3 or the empty vector with antibody against Pol II or TBP to examine the binding of these general transcription factors to the HIV-1 core promoter region. The normal IgG was used as a control. **(E)** The effects of miR-H3 on HIV-1 promoter activity without Tat expression. **(F)** Effect of miR-H3 on the activity of a TAR region deficient (deletion of 470 to 492 bp in HIV-1 5′ LTR) HIV-1 promoter. **(G)** The effect of miR-H3 on another retrovirus promoter. A RSV promoter-derived luciferase reporter construct was co-transfected with miR-H3 precursor or control vector, and the promoter activity was investigated by Dual-Luciferase assay. *P*-values were calculated using the two tailed unpaired Student’s t-test with equal variances, n = 3. *p < 0.05, ***p < 0.001.

With computational prediction, we surprisingly found a putative binding site of miR-H3 which covers the core promoter (the TATA box) in HIV-1 5′ LTR region (Figure 6A). The TATA box motif in HIV-1 5′ LTR starts two nucleotides further upstream and turns to the sequence CATATAA in all subtypes except for subtype E [36]. When mutations were introduced into the binding site in the TATA box region, the enhancement effect on promoter activity by miR-H3 was impaired (Figure 6B), suggesting that the direct binding between the core promoter and miR-H3 is required for its regulation. Furthermore, we mutated the TATA box region of CMV promoter to the same sequence as that of HIV-1 5′ LTR, and found that the transcription of this mutant could also be enhanced by miR-H3 (Figure 6C). These results suggest that the binding site in HIV-1 5′ LTR interacts with miR-H3 sequence-specifically and is required for the promoter activation induced by miR-H3. To investigate whether miR-H3 increases the binding of general transcription factors to the HIV-1 core promoter, we carried out ChIP assay with antibody against the RNA Polymerase II or the TATA box binding protein (TBP). The result suggested miR-H3 enhanced the association of both factors to the HIV-1 core promoter region (Figure 6D). As Tat protein is a very important regulatory factor for HIV-1 transcription, we investigated whether the interaction between Tat protein and TAR motif affected the HIV-1 promoter activation induced by miR-H3. Our data indicated that, in the absence of Tat, miR-H3 still upregulated HIV-1 promoter activity (Figure 6E). Alternatively, although the deletion of TAR significantly affected the promoter activity, the enhancement activity by miR-H3 was not affected (Figure 6F). Furthermore, miR-H3 did not affect the promoter activities of another retrovirus, Rous sarcoma virus (RSV), arguing against a non-specific transcription regulation on retroviruses (Figure 6G).

### The small RNAs targeting HIV-1 TATA box activate viral production from latently infected resting CD4^+^ T cells

**Figure 7:**
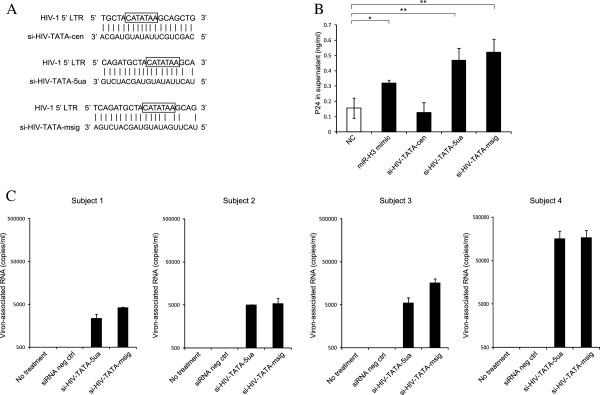
**Effect of small RNAs targeting HIV-1 TATA box upon HIV-1 production from resting CD4**^**+ **^**T cells isolated from HIV-1–infected patients on suppressive HAART. (A)** SiRNAs against HIV-1 TATA box were synthesized with the illustrated sequences. The TATA box motif was indicated with a box. **(B)** The miR-H3 miRNA mimic or siRNA was co-transfected with pNL4-3-deltaE-EGFP into HEK293T cells. The productions of P24 in the supernatant were determined with P24 ELISA at 48 hrs post-transfection. **(C)** Resting CD4^+^ T cells isolated from HIV-1-infected patients on suppressive HAART were transfected with control siRNA or si-HIV-TATAs. After 72 hrs, HIV-1 virions in the supernatants were collected and viral RNAs were isolated and amplified by real-time q RT-PCR with primer SK38 and SK39. The cutoff for virion-associated RNA is 500 copies per ml. *P*-values were calculated using the two tailed unpaired Student’s t-test with equal variances, n = 3. *p < 0.05, **p < 0.01.

HIV-1 latency in resting primary CD4^+^ T cells is the major barrier for the eradication of the viruses in HIV-1-infected patients on suppressive HAART. As miR-H3 has the enhancement activity on HIV-1 promoter, it is interesting to examine whether TATA-targeting miRNA/siRNA could be used to activate HIV-1 latency. To this end, we synthesized various small RNAs including miRNA mimics and siRNAs complementary to HIV-1 TATA box sequence. Among them, si-HIV-TATA-msig contains three nucleotides of mismatch in its 5′ end to avoid a possible RNA interference effects caused by the attachment of the small RNA to the 3′ LTR of HIV-1 mRNA (Figure 7A). We found that some siRNAs enhanced the promoter activity more efficiently than the synthesized miRNA mimics in HEK293T cells (with the HIV-pro-Luc plasmid, data not shown). When co-transfected with the Env-defective HIV-1 clone pNL4-3-deltaE-EGFP, siRNAs against HIV-1 TATA box potently increased the P24 antigen production with more stable performances (Figure 7B). Finally, we examined the effect of these small RNAs upon HIV-1 latency in resting CD4^+^ T cells directly isolated from HIV-1-infected individuals receiving suppressive HAART. Post-integration HIV-1 latency in these cells was confirmed by the detection of integrated HIV-1 proviruses in the chromosomal DNA via Alu-PCR (Additional file 1: Figure S6). Stimulation with anti-CD3/anti-CD28 induced the production of a large number of viral particles (Additional file 1: Figure S7). These data suggest that the primary resting CD4^+^ T-lymphocytes isolated from the patients were latently infected. After transfection with the siRNAs complementary to HIV-1-TATA box, these resting CD4^+^ T cells generated significantly more HIV-1 particles than those treated with a negative control siRNA (Figure 7C). This data indicated that a siRNA complementary to HIV-1 TATA box alone is able to activate HIV-1 transcription in the latently-infected resting CD4^+^ T-lymphocytes.

## Discussion

In this report, we found that a novel HIV-1-encoded miRNA could upregulate its viral transcription by targeting the TATA box in the 5′ LTR. Several studies have revealed that small non-coding RNAs (e.g. miRNA and siRNA) target to gene promoters are able to induce gene transcription activation or silence. For instance, miR-373 can activate the expression of E-cadherin and cold-shock domain-containing protein C2 (CSDC2) through a target site in their promoters [15]. Another miRNA, miR-423-5p, induces transcriptional silencing by targeting a highly conserved region in the promoter of progesterone receptor (PR) gene [16]. At the same time, the potential of synthetic small RNAs to manipulate gene transcription was also explored. Morris and coworkers initially reported the inhibition of the EF1α promoter with a siRNA targeting approximately 100 bp upstream the EF1α transcription start site (TSS) [37]. Several studies subsequently reported that small RNAs could also induce transcriptional gene activation by targeting gene promoters [38,39]. These reports indicate the existence of a small RNA guided transcription regulation mechanism in the nucleus of mammalian cells. For the first time, our study revealed that viruses have exploited this host mechanism to regulate viral replication by virus-encoded miRNAs. However, the targeting sites of miRNA or siRNA on gene promoter in previous studies distribute in a wide range (~1000 bp upstream of the TSS). In this study, miR-H3 targets the key position for transcription initiation -TATA box, wherein the polymerase II pre-initiation complexes (PICs) are assembled. This finding raises the possibility that miRNAs directly participate in the transcription initiation regulation in mammalian cells.

The transcription activity of HIV-1 provirus is finely modulated in different host cells, which is accomplished through a lot of cellular transcription factors and viral proteins. Cellular transcription factors such as Sp1, NF-κB, NF-AT, LEF-1/TCF-1α, C/EBP, and CREB are important for the activation of HIV-1 LTR-driven transcription [40-49]; Conversely, cellular factors including LBP-1, TDP-43, YY1 and P53 exhibit inhibitory effects on LTR-driven transcription [50-54]. It is noteworthy that the effects of many cellular factors are cell-type dependent and there are complicated interaction among these factors (more discussion see the review of Rohr et al. [55]). A key viral regulator of HIV-1 transcription activity is the regulatory protein-Tat, which is produced during early phase of infection and binds to the trans-activation-responsive region (TAR) located at the 5′-end of viral mRNAs [56]. After binding, Tat recruits a diverse series of transcriptional complexes to the viral promoter and activates transcription activity [57]. These complexes include enzymes with histone and factor acetyl transferase (HAT and FAT respectively) activities, which modify chromatin conformation at the proviral integration site [58], and a protein complex (P-TEFb) that hyper-phosphorylates the carboxy-terminal domain (CTD) of RNA polymerase II, thus promoting the initiation and elongation of viral transcription [59,60]. Vpr and Nef also have effects on HIV-1 transcription, which through the interaction with Tat or up-regulating the expression of activating factors such as NF-AT, NF-κB, and AP-1 [61-64]. In this study, we showed that miR-H3 is another HIV-1- encoded *cis*- regulatory element, in addition to Tat, that direct interacts with viral element and regulates transcription activity. It is interesting to investigate whether the effect of miR-H3 is cell-type dependent and identify its cellular co-factors. Our findings further reveal the complexity of transcription regulation for HIV-1.

The advances in next-generation sequencing technology have greatly fueled the discovery of small RNAs, especially the low expressed miRNAs. However, their functions are largely unexploited. One opinion about these low expressed miRNAs is that they may not have function due to the low expression levels. This idea is reasonable according to the well-known paradigm that miRNAs function in cytoplasm through targeting the 3′ UTR of mRNA for translation repression. This model requires a considerable amount of miRNAs for their functions since their targets are relatively highly expressed. But our data suggest miR-H3 and many cellular miRNAs target the core promoter of HIV-1 virus and many important genes (unpublished data), which are on the chromosomal DNA with very limited copy number in the nucleus in contrast to the massive mRNA molecules in the cytoplasm. Thus the requirement of the accumulation level of these TATA box targeting miRNAs is relatively low. Our data and previous studies on promoter targeting miRNAs probably provide a novel function model for the low expressed miRNAs.

Latent infection of HIV-1 is the major barrier for the eradication of the viruses in patients on suppressive HAART. The first step to remove latent viral reservoirs is reactivating the latent proviruses. Several approaches have been developed to activate latent virus transcription including activating T lymphocytes with IL-2 or IL-2 plus anti-CD3/anti-CD28 antibody [65,66], protein kinase C (PKC) activators (e.g. prostatin [67]), and activating transcription with small molecule inhibitors of histone deacetylases without inducing host cell activation (such as, valproic acid (VPA), suberoylanilide hydroxamic acid (SAHA))[68-70]. However, the first approach has been shown to cause serious toxic effects, and the latters are speculated about causing global gene expression activation with unpredictable side effects. Thus, a HIV-1 provirus specific activating reagent is ideal for purging the latent reservoir. In this study, we demonstrated a HIV-1 encoded miRNA could activate HIV-1 transcription in a sequence-specific manner, and the synthesized small RNA induced viral production from resting CD4^+^ T cells from patients receiving suppressive HAART treatment. Together with our previous finding that some cellular miRNAs have contributed to the latency of HIV-1[20], a combination of the small RNA(s) targeting to HIV-1 TATA box and the inhibitors of these cellular miRNAs will provide a HIV-1 specific approach for eradicating HIV-1 latent reservoir more safely.

## Conclusions

In this study, we identified a novel HIV-1-encoded miRNA miR-H3, which potently enhances viral production. Unlike most miRNAs that target the 3′ UTR of mRNA for translation repression, miR-H3 targets the TATA box in HIV-1 5′ LTR to upregulate the promoter activity. It represents another HIV-1-encoded element, in addition to Tat, that activates viral transcription via *cis* regulation. These findings reveal a new layer of HIV-1 replication regulation and may serve as the basis for an innovative approach to specifically activate latent infected HIV-1 viruses.

## Methods

### Ethics statement

This research was approved by the Ethics Review Board of The Eighth People’s Hospital at Guangzhou (Guangzhou Infectious Disease Hospital, Guangzhou, China) and the Ethics Review Board of Sun Yat-Sen University. HIV-1-infected patients were recruited at The Eighth People’s Hospital at Guangzhou and given written informed consent with approval of the Ethics Committees. De-identified human peripheral blood mononuclear cells (PBMCs) from healthy blood donors were obtained from local volunteers. We did not have any interaction with these human subjects or protected information, and therefore no informed consent was required.

### MiRNA *in silico* prediction

The genomic sequence of pNL4-3 was downloaded from NCBI and submitted to web server of mireval (http://mimirna.centenary.org.au/mireval/). A total of 9 miRNA candidates including two associated with TAR regions were suggested. These candidates were further filtered by comparing their secondary structure with that of HIV-1 genomic RNA [31] and coincident ones were chosen for experimental validation. The miRNA precursor structure prediction was performed with the Mfold webserver [71]. Representative sequences of major HIV-1 subtypes were downloaded from the HIV sequence database (http://www.hiv.lanl.gov/content/sequence/HIV/mainpage.html). Alignment of these sequences was performed with Clustal X program. The miRNA binding sites on HIV-1 were predicted with RNA-hybrid web server (http://bibiserv.techfak.uni-bielefeld.de/rnahybrid).

### Cell culture

Sup-T1 and HEK293T cells were obtained from ATCC (American Type Culture Collection, Manassas, VA) and cultured according to ATCC recommendations. TZM-bl cells were obtained from the AIDS Research and Reference Reagent Program, NIAID, US NIH. Human PBMCs were isolated from the whole blood of healthy donors by Ficoll-Hypaque Solution (HAO YANG, Tianjin, China). The resting primary CD4^+^ T lymphocytes were then isolated from PBMCs with CD4^+^ T Cell Isolation Kit II (BD). Human primary PBMCs and CD4^+^ T cells were grown in the RPMI 1640 conditioned media supplemented with 10% fetal bovine serum (FBS), 50 U/ml penicillin and 50 μg/ml streptomycin.

### Plasmids, siRNAs and antibodies

The infectious HIV-1 clone (pNL4-3) and Env-defective HIV-1 clone (pNL4-3-deltaE-EGFP) were obtained through the AIDS Research and Reference Reagent Program, NIAID, US NIH. The precursor of miR-H3 and hsa-miR-150 were amplified by PCR and directionally cloned into the downstream of the EGFP gene in the pEGFP-C1 vector (BD Biosciences). The pNL4-3-miR-H3MT plasmid was constructed by introducing mutations in the region for miR-H3-3p mature miRNA without changing amino acid code. Similar mutations were also introduced into non-infectious HIV-1 clone, pNL4-3-deltaE-EGFP. HIV-pro-Luc plasmid was constructed by replacing the promoter of Luciferase gene in the pMIR-REPORT Luciferase vector (Invitrogen) with HIV-1 5′ LTR sequence. MMLV-Luc-HIV_3LTR plasmid was constructed by replacing the promoter of Luciferase gene in the pMIR-REPORT vector with the MMLV promoter and inserting the HIV-1 3′ LTR downstream the luciferase gene. Several mutations were introduced into miR-H3 binding site in the 5′ LTR sequence. HIV-pro-Luc-mtTAR plasmid was constructed with deleting 5′ half region of TAR motif (470 bp to 492 bp region in HIV-1 5′ LTR) to abolish its functional secondary structure. RSV-pro-Luc plasmid was constructed by replacing the promoter of Luciferase gene in the pMIR-REPORT vector with the RSV promoter. All the constructs were verified by sequencing. The siRNAs against Drosha and Exportin-5 genes were purchased from Dharmacon. Anti-β-actin antibody (D6A8) was purchased from CST (Danvers, MA). Anti-human CD3 and anti-human CD28 antibodies were from BD (Palo Alto, CA). The rabbit anti-P24 antibody was prepared by our lab.

### Infection and transfection

Infectious HIV-1 clone pNL4-3 was transfected into 60% confluent HEK293T cells (100 mm plate) using Lipofectamine 2000 according to the manufacturer’s protocol (Invitrogen). Viral supernatant was collected 2 days after transfection and viral production was determined by P24 ELISA kit. Five ng P24 f infectious HIV-1 viruses were used to infect 2X 10^6^ activated human CD4^+^ T lymphocytes for 3 hrs at 37°C. The cells were then washed three times with cold PBS and add fresh conditioned medium containing IL-2 (10 ng/ml). Supernatants of cell culture were collected in 2-3 days interval and subject to P24 ELISA detection. Transfection of HEK293T and TZM-bl cells was performed with Lipofectamine 2000 (Invitrogen) according to the manufacturer’s protocol. Transfection of primary CD4^+^ cells with small RNA was performed with RNAiMAX (Invitrogen) according to the manufacturer’s protocol.

### Quantitative real-time RT–PCR analysis

Total RNA from HEK293T or CD4^+^ T cells was isolated with Trizol reagent (Invitrogen) and then subjected to cDNA synthesis using PrimeScript RT reagent Kit (Takara). All primers were annealed at 37°C and RT was processed at 42°C. Quantitative PCR was performed with SYBR Premix ExTaq II Kit (Takara) by following the manufacturer’s instructions. The expressions of HIV-1 total RNAs were determined with the primer pair HIVTotRNA-5 F/R. The HIV-1 RT activity assay was performed by following the method described in Vermeire et al. [72]. An in vitro-synthesized HIV-1 RNA, after quantification [73], was used as the external control for measuring virion-associated viral RNA. Quantification was normalized to the housekeeping gene U6 or β-actin. All primers for HIV-1 gene detection were listed in the additional files. The relative expression levels were calculated using the following equation: *A* = 2^[*Ct*(ref) − *Ct*(ref ‒ control)] − [*Ct*(sample) − *Ct*(sample ‒ control)]^.

### Dual-luciferase reporter assay

HEK293T cells were seeded in 48-well plates (Corning) at a density of 20,000 cells per well one day before transfection. One to 5 ng of HIV-1 wildtype or mutated promoter driven-firefly luciferase (FL) reporter and 2 ng renilla luciferase (RL) constructs were co-transfected with miRNA precursor/mock control into HEK293T cells using Lipofectamine 2000 (Invitrogen) by following the manufacturer’s protocol. After 24-48 hrs, FL and RL activities were measured with the Dual-Glo luciferase assay system according to the manufacturer’s instructions (Promega).

### Protein analysis

Infectious or defective viral particle production in cell cultures was determined with P24 ELISA kit by following the manufacturer’s protocol. Western blotting was carried out as described previously with some minor modifications [74]. The anti-P24 or anti-β-actin antibodies were used to detect HIV-1 P55, P41 and P24 or β-actin protein respectively.

### RNase protection assay (RPA)

The activated CD4^+^ T-cells were infected with viruses produced from HIV-1 clone pNL4-3 or mock infected. At 48 hrs post infection, the fresh CD4^+^ T-cells from the same donor were co-cultured with the infected CD4^+^ T-cells with a ratio of 3:1 for another 72 hrs. Then the total RNAs were isolated with Trizol Reagent (Invitrogen) and the small RNAs (<200 nt) were enriched by using the mirVana miRNA isolation kit (Ambion). The procedure of RPA described by Gilman was followed with some minor modifications [75]. The RNA probe was a sequence complementary to nt 34/71 of miR-H3 precursor and was synthesized from DNA templates by in vitro transcription using the T7 RNA polymerase (NEB) and radiolabeled by random incorporation of α-^32^ P UTP (Perkin Elmer). The radiolabeled probe was hybridized with 10 ug of small RNA overnight at 45°C. After the hybridized RNAs were then treated with RNases A/T1, the protected RNAs were separated by denaturing PAGE (12%) and visualized by autoradiography.

### Chromatin immunoprecipitation (ChIP) assay

ChIP assay was performed with Magna ChIP™ A/G Kit (Millipore) by following the manufacture’s instruction. Briefly, HEK293T cells were co-transfected with pNL4-3-deltaE-EGFP and pre-miR-H3 or the empty vector. At 48 hrs post transfection, the cells were collected to carry out ChIP assay with anti-Pol II (8WG16, Covance), TBP (Abcam), or normal IgG antibodies. The HIV-1 promoter sequence corresponding to -50 - (+)50 bp relative to the TSS was detected with qPCR.

## Competing interests

The authors declare that they have no competing interests.

## Authors’ contributions

YZ conducted the most experiments and performed the analyses. YZ, MF, and HZ wrote the manuscript. YZ and HZ conceived the study and contributed to data interpretation, and HZ supervised the project. MF, GG, BL, ZH, HL performed some experiments. JZ, XG and WC contributed to collect the clinical samples. All authors read and approved the final manuscript.

## Supplementary Material

Additional file 1: Figure S1 The genuine secondary structure of miR-H3 precursor corresponding sequence in HIV-1 genome by Watts et al [31]. **Figure S2.** Primer extension assay of miR-H3-3p. Total RNAs were isolated from HEK293T cells transfected with a lentiviral vector pCMV-ΔR8.2 which contains the miR-H3 precursor or a control plasmid for 48 hrs. A small RNA band was detected only in the lane of pCMV-ΔR8.2 transfection by a probe specific to miR-H3-3p sequence. **Figure S3.** Ectopic expression of miR-H3 by constructs containing its wildtype or mutated precursors. Top, the mutated nucleotides were indicated in red; bottom, mature miR-H3-3p sequence was tested with real-time qPCR and normalized to U6. The empty vector was transfected as a control. **Figure S4.** The effect of miR-H3 on integrated HIV-1 reporter system. TZM-bl cells, containing an integrated HIV-1 promoter-driven luciferase cassette in chromosomal DNA, were transfected with the construct harboring miR-H3 precursor or an empty vector. The transcription activities of HIV-1 promoter were examined by luciferase assay. **Figure S5.** MiR-H3-3p processed from mutated pNL4-3-deltaE-EGFP (A) or pNL4-3 constructs (B). The plasmid were transfected into HEK293T cells, 48 hrs later total RNAs were isolated and miR-H3-3p expression was determined with qRT-PCR and normalized to U6. **Figure S6.** Confirmation of integrated HIV-1 proviruses in the chromosomal DNA from resting CD4^+^ T cells isolated from HIV-1-infected patients on suppressive HAART using Alu-PCR. **Figure S7.** The virus production was induced from resting CD4^+^ T cells isolated from HIV-1-infected patients on suppressive HAART by anit-CD3/anti-CD28. The viral production in the supernatant was measured by HIV-1 P24 ELISA. Click here for file
