# Supplementary material for: A novel HIV-1-encoded microRNA enhances its viral replication by targeting the TATA box region
Source: Retrovirology. 2014 Mar 12;11:23. doi: 10.1186/1742-4690-11-23 (PMC4007588; doi:10.1186/1742-4690-11-23)
Supplement: Additional file 1: Figure S1 — The genuine secondary structure of miR-H3 precursor corresponding sequence in HIV-1 genome by Watts et al [31]. Figure S2. Primer extension assay of miR-H3-3p. Total RNAs were isolated from HEK293T cells transfected with a lentiviral vector pCMV-ΔR8.2 which contains the miR-H3 precursor or a control plasmid for 48 hrs. A small RNA band was detected only in the lane of pCMV-ΔR8.2 transfection by a probe specific to miR-H3-3p sequence. Figure S3. Ectopic expression of miR-H3 by constructs containing its wildtype or mutated precursors. Top, the mutated nucleotides were indicated in red; bottom, mature miR-H3-3p sequence was tested with real-time qPCR and normalized to U6. The empty vector was transfected as a control. Figure S4. The effect of miR-H3 on integrated HIV-1 reporter system. TZM-bl cells, containing an integrated HIV-1 promoter-driven luciferase cassette in chromosomal DNA, were transfected with the construct harboring miR-H3 precursor or an empty vector. The transcription activities of HIV-1 promoter were examined by luciferase assay. Figure S5. MiR-H3-3p processed from mutated pNL4-3-deltaE-EGFP (A) or pNL4-3 constructs (B). The plasmid were transfected into HEK293T cells, 48 hrs later total RNAs were isolated and miR-H3-3p expression was determined with qRT-PCR and normalized to U6. Figure S6. Confirmation of integrated HIV-1 proviruses in the chromosomal DNA from resting CD4+ T cells isolated from HIV-1-infected patients on suppressive HAART using Alu-PCR. Figure S7. The virus production was induced from resting CD4+ T cells isolated from HIV-1-infected patients on suppressive HAART by anit-CD3/anti-CD28. The viral production in the supernatant was measured by HIV-1 P24 ELISA. [file 1742-4690-11-23-S1.docx]

Additional files for

**A Novel HIV-1-Encoded MicroRNA Enhances Its Viral Replication by Targeting the** **TATA Box Region**

Yijun Zhang, Miaomiao Fan, Guannan Geng, Bingfeng Liu, Zhuoqiong Huang, Haihua Luo, Jie Zhou, Xuemin Guo, Weiping Cai

and Hui Zhang*

This file includes:

Supplemental Data (Fig. S1 to S7)

Supplemental Material and Methods

Supplemental References


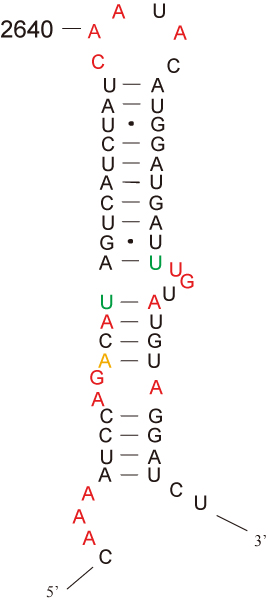


**Fig.S1** The genuine secondary structure of miR-H3 precursor corresponding sequence in HIV-1 genome by Watts et al. [31].


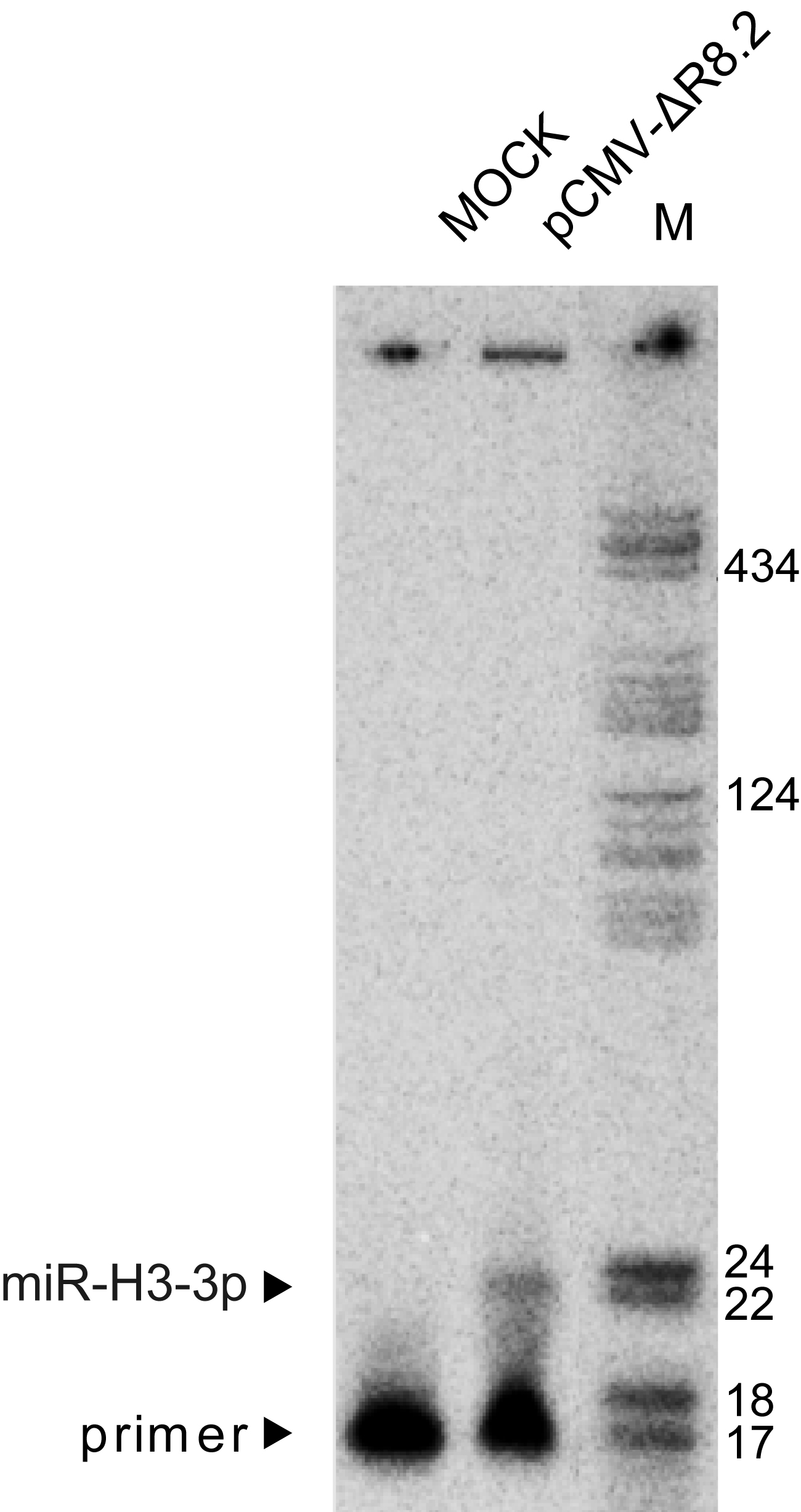


**Fig. S2** Primer extension assay of miR-H3-3p. Total RNAs were isolated from HEK293T cells transfected with a lentiviral vector pCMV-ΔR8.2 which contains the miR-H3 precursor or a control plasmid for 48 hrs. A small RNA band was detected only in the lane of pCMV-ΔR8.2 transfection by a probe specific to miR-H3-3p sequence.


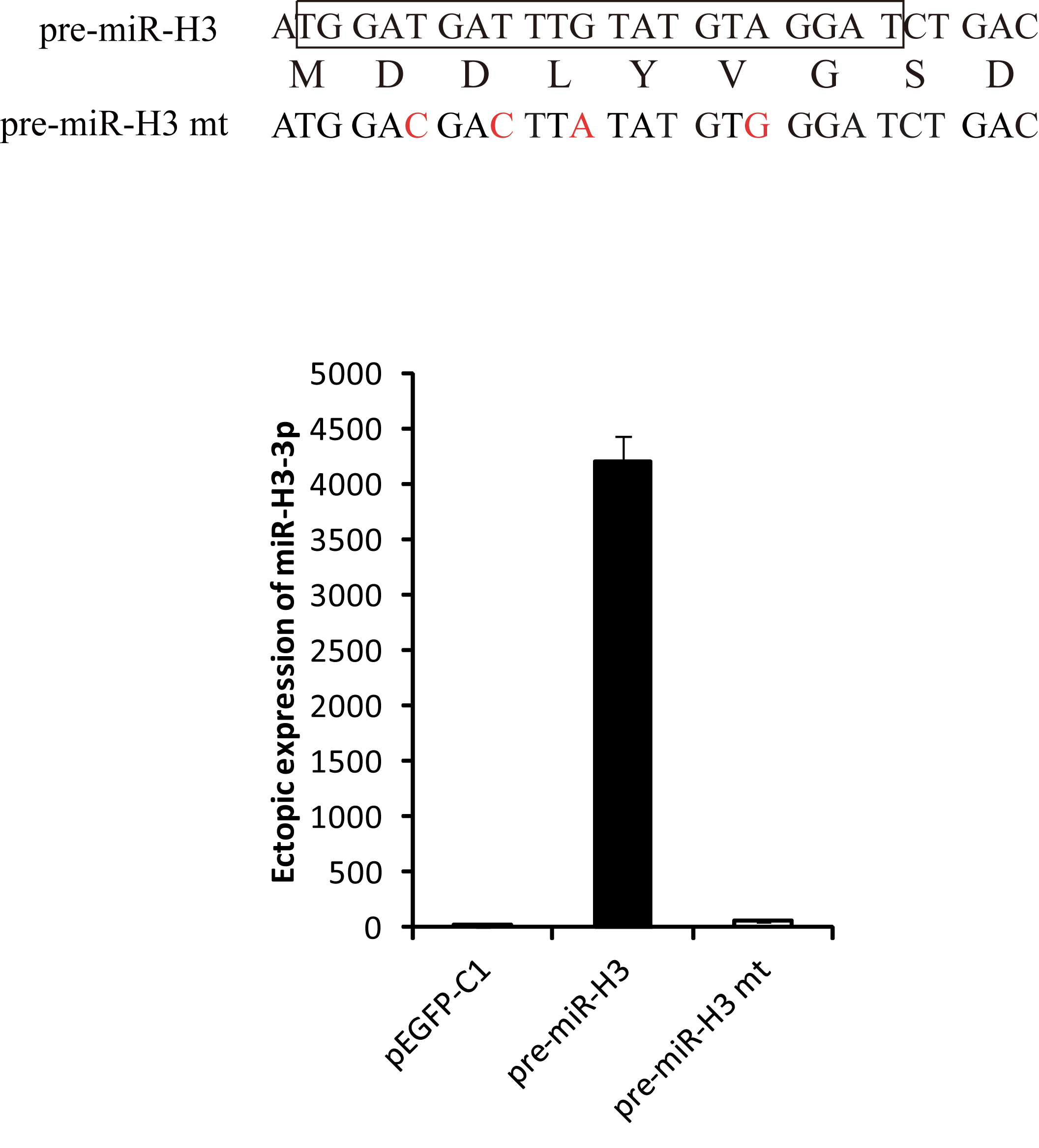


**Fig. S3** Ectopic expression of miR-H3 by constructs containing its wildtype or mutated precursors. Top, the mutated nucleotides were indicated in red; bottom, mature miR-H3-3p sequence was tested with real-time qPCR and normalized to U6, an empty vector was transfected as a control.


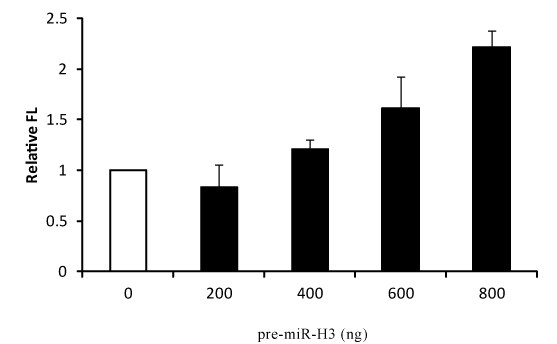


**Fig. S4** Effect of miR-H3 on integrated HIV-1 reporter system. TZM-bl cells, containing an integrated HIV-1 promoter-driven luciferase cassette in chromosomal DNA, were transfected with the construct harboring miR-H3 precursor or an empty vector. The transcription activities of HIV-1 promoter were examined by luciferase assay.


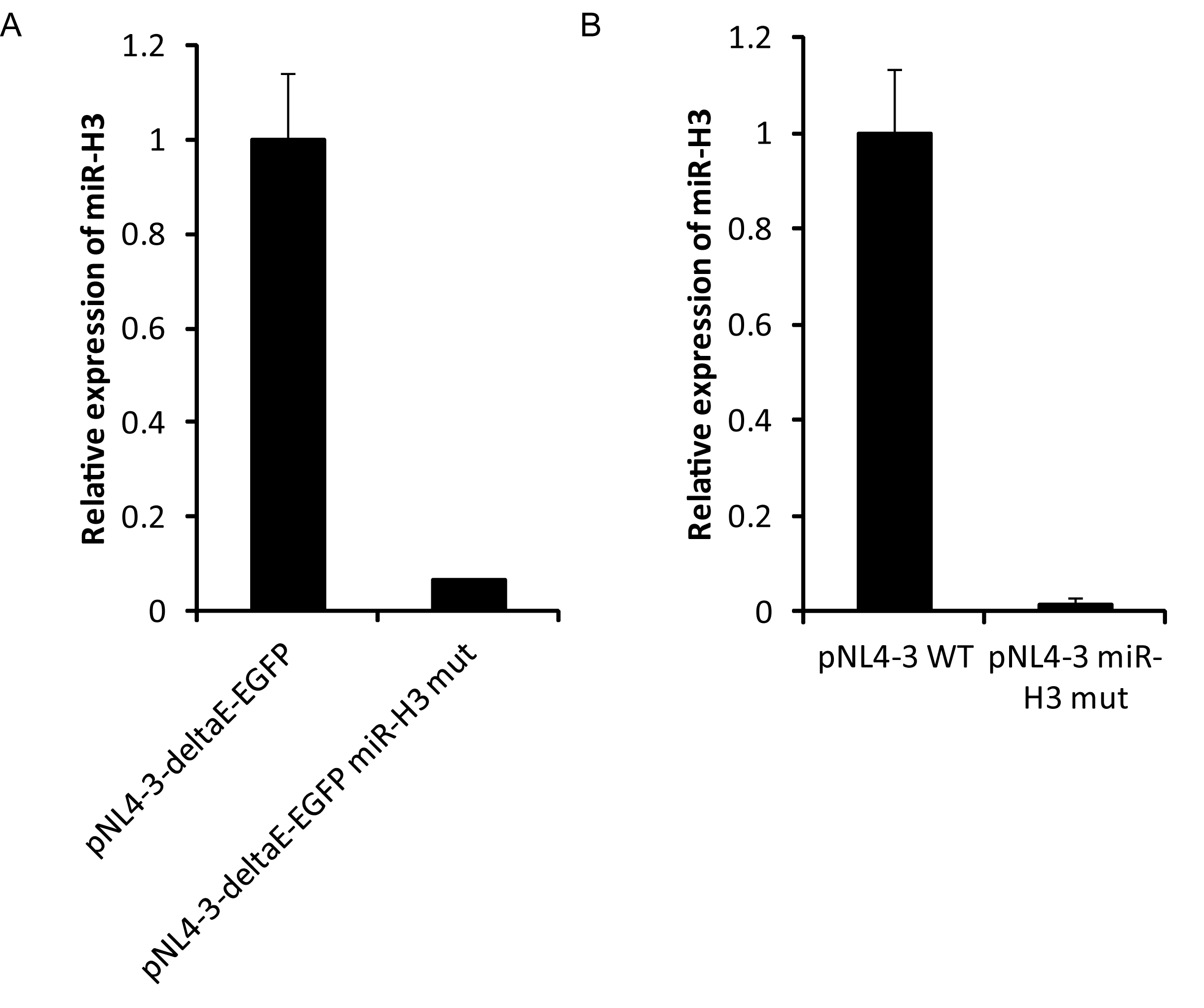


**Fig. S5** MiR-H3-3p processed from mutated pNL4-3-deltaE-EGFP (**A**) or pNL4-3 constructs (**B**). The plasmid were transfected into HEK293T cells, After 48 hrs total RNAs were isolated and miR-H3-3p expression was determined with qRT-PCR and normalized to U6.


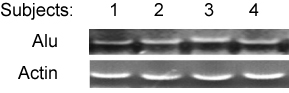


**Fig. S6** Confirmation of integrated HIV-1 proviruses in the chromosomal DNA from resting CD4^+^ T cells isolated from HIV-1-infected patients on suppressive HAART using Alu-PCR.

**
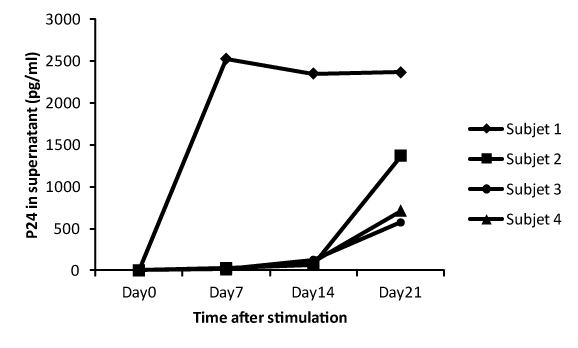
**

**Fig. S7** Virus production was induced from resting CD4^+^ T cells isolated from HIV-1-infected patients on suppressive HAART by anit-CD3/anti-CD28. The viral production in the supernatant was measured by HIV-1 p24 ELISA.

**Supplemental Methods**

**The primer sequences were designed as follows:**

**Quantitative real-time RT–PCR analysis**

SK38, 5’-ATAATCCACCTATCCCAGTAGGAGAAA-3’;

SK39, 5’-TTTGGTCCTTGTCTTATGTCCAGAATGC-3’;

HIVTotRNA-5F, 5’-CTGGCTAACTAGGGAACCCACTGCT-3’;

HIVTotRNA-5R, 5’-GCTTCAGCAAGCCGAGTCCTGCGTC-3’;

β-actin-F, 5’-GCATGGAGTCCTGTGGCA-3’;

β-actin-R, 5’-CAGGAGGAGCAATGATCTTGA-3’;

GAPDH-F, 5’-TGCACCACCAACTGCTTAGC-3’;

GAPDH-R, 5’- GGCATGGACTGTGGTCATGAG-3’;

miR-H3-3p-F, 5’-GCGGCGGTGGATGATTTGTA-3’;

miR-H3-3p-RT, 5’- GTCGTATCCAGTGCAGGGTCCGAGGTATTCGCACTGGATACGACCCTACA-3’;

miR-H3-5p-F, 5’-GCGGCGGAAATCCAGACATAGTC-3’;

miR-H3-5p-RT, 5’- GTCGTATCCAGTGCAGGGTCCGAGGTATTCGCACTGGATACGACATAGAT-3’;

UNIREVERSE, 5’-GTGCAGGGTCCGAGGT-3’;

**Quantitative real-time PCR for ChIP assay**

HIV5LTRsense398, 5’-TGGGGAGTGGCGAGCCCTCAGATGC-3’;

HIV5LTRantisense493, 5’-GCAGTGGGTTCCCTAGTTAGCCAGA-3’;

**Primer extension assay**

Total RNAs were isolated from HEK293T cells transiently transfected with pCMV-ΔR8.2 or a control plasmid and harvested 48 hrs later. Primer extension assay was then performed as described previously with some minor modifications [26]. Briefly, 10 μg RNA were hybridized with 5’ radiolabeled DNA oligo-nucleotide complementary to miR-H3-3p and allowed for 1 hr extension at 42°C. The extended primers were separated by denaturing PAGE (15%) and visualized by autoradiography. The probe used to detect miR-H3-3p is 5’ -gatcctacatacaaatc- 3’.

**Alu-PCR.**

Genomic DNA was extracted from the resting CD4+ T cells isolated from HIV-1–infected individuals. The integrated HIV-1 was first amplified using primer pairs specific to Alu fragments and HIV-1 U3 sequence,, followed by re-amplification with primer pairs within U3 region which are at the upstream of the U3 3’- primer for the first amplification. The primer pairs and procedures described previously was followed [[76](#_ENREF_1)].

**REFFERENCES:**

76. Butler SL, Hansen MS, Bushman FD: **A quantitative assay for HIV DNA integration in vivo.** *Nat Med* 2001, **7:**631-634.
